# Nanoparticle Platforms for Antigen-Specific Immune Tolerance

**DOI:** 10.3389/fimmu.2020.00945

**Published:** 2020-05-20

**Authors:** Edward B. Thorp, Christian Boada, Clarens Jarbath, Xunrong Luo

**Affiliations:** ^1^Departments of Pathology & Pediatrics at Northwestern University Feinberg School of Medicine, Chicago, IL, United States; ^2^Division of Nephrology, Department of Medicine, Duke University School of Medicine, Durham, NC, United States; ^3^Duke Transplant Center, Duke University School of Medicine, Durham, NC, United States

**Keywords:** nanoparticles, tolerance, transplant, rejection, immunity

## Abstract

Innovative approaches in nanoparticle design have facilitated the creation of new formulations of nanoparticles that are capable of selectively calibrating the immune response. These nanomaterials may be engineered to interact with specific cellular and molecular targets. Recent advancements in nanoparticle synthesis have enabled surface functionalization of particles that mimic the diversity of ligands on the cell surface. Platforms synthesized using these design principles, called “biomimetic” nanoparticles, have achieved increasingly sophisticated targeting specificity and cellular trafficking capabilities. This holds great promise for next generation therapies that seek to achieve immune tolerance. In this review, we discuss the importance of physical design parameters including size, shape, and biomimetic surface functionalization, on the biodistribution, safety and efficacy of biologic nanoparticles. We will also explore potential applications for immune tolerance for organ or stem cell transplantation.

## Introduction

**Figure 1:**
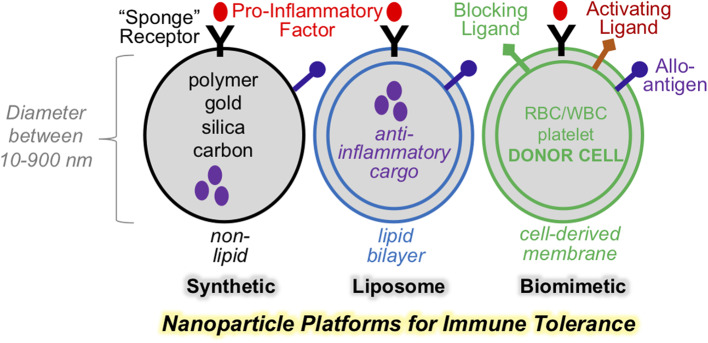
Nanoparticle platforms for various designs for immune regulations. Selection nanoparticle physical properties is key to effective therapeutic targeting. Synthetic and liposome-based nanoparticle may be engineered to display surface ligands, as well as encapsulate bioactive cargo. Biomimetic nanoparticle incorporate cell-derived membranes and heterogenous composition of surface and functional ligands with the capacity to avtive or block signaling on cellular targets.

Nanoparticles have long been applied in medicine as drug delivery and diagnostic imaging agents. This approach has proven efficacy in targeted delivery of therapeutic compounds, including synthetic, recombinant, and genetic materials. To achieve targeted delivery, nanoparticles are formulated using design principles that are grouped into two principle categories: passive targeting and active targeting. The first category, “passive targeting,” refers to approaches that manipulate physical parameters of nanoparticle size, shape, surface charge, stiffness, pH, and material composition to alter nanoparticle pharmacodynamics, therapeutic range (1, 2), their targeting to specific tissue types and safety profile (3). The second category, or “active targeting,” refers to surface modifications of nanoparticles, such as the incorporation of whole cell membranes or native and synthetic proteins. This approach is referred to as the “biomimetic design” (Figure 1). The hallmark of biomimetic design is particle surface modification with cell surface-like components. This endows the nanoparticle with more sophisticated functions that include cell targeting, particle transmigration, and more recently, activation of molecular signaling. This last feature, provides the opportunity to develop platforms with the capacity to regulate specific immune signaling axes. Indeed, biomimetic nanoparticles represent a future of nanomedicine and take advantage of the natural propensities of cell-surface ligands to regulate cell crosstalk and cell activation. Such an approach has the potential to incorporate the best of cell-based therapies, but with reduced cost and enhanced particle homogeneity. In addition, biomimetic approaches may inherently replicate ligand properties and varieties that cannot be fully captured by synthetic platforms. This is particularly important in strategies intended for suppressing complex immune reactivities to self- or allo-antigens. Below, we will discuss physical design principles of nanoparticles for the purpose of immune modulation. We also focus on biomimetic designs and their exploitation to enhance immunomodulation. These principles will be discussed in the context of therapeutic platforms for immune tolerance for organ or stem cell transplant.

## Section 1. Physical Parameters of Nanoparticles that Govern Their Utility for Immune Modulation

When formulating nanoparticles for targeting cellular pathways, it is important to consider the contribution of physical parameters to particle bio-distribution and cell internalization. For example, there exist specific characteristics of intravenous injectable particles that are key to delivering antigens to immunomodulatory cells. These traits are distinct from other nanotechnology-based platforms such as sub-dermal injectables or implants. A good starting point for these structural formulations is the consideration of sequential pathways and barriers that must be navigated from the site of injection to the target tissue. When nanoparticles are first injected into the blood stream, an initial path of particle navigation is through the vascular tree and therefore specific features of the target organ must be considered. These include organ vascularity, vascular luminal size, and blood flow velocity within the target organ. Poorly vascularized organs will serve as poor targets if blood is the conduit. The second parameter, luminal size, determines the effective size range of the nanoparticle *per se*. This becomes increasingly critical within smaller capillaries. Blood flow velocity is also an important consideration, as higher velocities in larger vessels tend to propel nanoparticles forward, whereas slower velocities (such as in capillaries or in larger vessels with aberrant geometry) tend to promote nanoparticles to marginalize. In some cases, the pH of the extracellular milieu must also be considered. For example, for oral route administration, nanoparticles will be exposed to a wide variation of tissue acidity. Additional physical parameters such as the chemical reactivity of nanoparticle materials, particle shape, and surface charge, have all been exploited to achieve the desired biodistribution and application in disparate organs and cellular compartments (1, 2). The combinatorial potential of modifiable platforms remains an area of active research and offers a vast range of opportunities to formulate novel platforms for specific applications. Given the wide spectrum of engineerable parameters, it is important to have a grasp of the toolset available for formulating specific platforms for minimizing off-target effects while maximizing effective modulation of the immune system. We will discuss individual parameters below.

## Nanoparticle Size for Immune Modulation

One of the most important parameters to consider when designing nanoparticles is particle size. Size will impact how particles behave in the bloodstream as well as how they are internalized by cells and how they are transported once inside a cell.

Studies using nanoparticles for intravenous delivery converge toward an ideal size range between 30 and 300 nm to maximize particle circulation. Theoretical studies analyzing the forces acting upon nanoparticles in the bloodstream generally support these observations, favoring toward smaller particles as they are less prone to marginalize in normal laminar flow (4–8). A further refinement to the above view by additional *in vivo* observations (9, 10) is that the ideal balance between prolonged circulation and desired biodistribution lies somewhere around 100–200 nm (11, 12), and that smaller particles of <20 nm may be quickly cleared through kidney filtration.

At the organ level, nanoparticles with a diameter of 200 nm and above predominantly accumulate in the red pulp of the spleen (13) which allow them to preferentially interact with immune cells at that location. Once nanoparticles adhere to the vascular endothelium, they next extravasate, either through passive or active processes, and come into contact with the extracellular matrix. At the cellular level, all particles will eventually be engulfed by phagocytes. However, strategies to inhibit phagocyte internalization have also been developed, such as incorporation of CD47, a marker of self, on nano-platforms (2). Particles <1 micrometer in diameter are more readily internalized than larger particles (14). Safety concerns, such as potential occlusion of lung capillaries, are typical more common when particle diameters are >1 micrometer; therefore particles larger than this threshold are in general not used (15). In reviewing published literature for preparation of this manuscript, only a few reports were found on the toxicity of microparticles particles 1 micrometer and above, as injecting nanoparticles above this size has customarily been avoided due to concerns of embolism (16). An exception are microparticles with large surface areas and drug loading capacity such as porous silicon particles, which have been exploited for prolonged release of therapeutic cargo as they degrade within implants (17) or as injectables (18). It has been speculated that avid Internalization by phagocytic cells of larger particles is often a hinderance to their specific targeting, as many would be cleared from the blood stream before reaching their target organs/tissues. On the other end of the spectrum, as particles become smaller, their ability to accumulate within cells increases while their propensity for clearance from the cell decreases. Prolonged accumulation renders smaller nanoparticles toxic to the host cell. *In vitro* studies with smaller nanoparticles such as gold (19) or quantum dots (QD) (20) have revealed that cytotoxicity is dependent on nanoparticle size. For instance, smaller uncoated cadmium telluride (CdTe) QDs become cytotoxic at a concentration of 1 μg/mL, with cell death characterized by chromatin condensation and membrane blebbing. Similarly, a study of gold nanoparticles with a core diameter of 2 nm found these particles to be toxic at higher concentrations with evidence of cellular membrane disruption (3). A separate consideration for particles with a hydrodynamic diameter (diameter of particles once hydrated) smaller than 5.5 nm is their immediate excretion by the kidney (21, 22) where filtration slits of 4–6 nm in width are found in the epithelial lining (23).

At the cellular level, pathways for nanoparticle internalization are also dependent on particle size which in turn determines particle subcellular destination. Studies on the impact of nanoparticle size on cellular internalization pathways indicate that cells can internalize particles up to 500 nm in any given dimension. Generally, internalization of nanoparticles is mediated by classical non-specific internalization pathways such as macropinocytosis, clathrin-mediated, and caveolin-mediated endocytosis (24). Particle internalization via immune cell scavenger receptors has also been described (25, 26). Upon encountering with cells, cationic liposomes below a diameter of 500 nm are internalized by dendritic cells through caveolae-mediated non-degrative endocytosis. In contrast, larger lipoplexes (~500 nm diameter or greater) have been shown to be taken up by dendritic cells via clathrin-mediated endocytosis and micropinocytosis, leading to a lysosomal degradation pathway (1).

Once internalized, intracellular directional transportation and, in the case of antigenic cargos, their subsequent surface presentation, are also dependent on particle size. Small particles (20–200 nm) heavily rely on microtubules and clathrin-coated pits for cellular transport. Two hundred newton meters appears to be the threshold above which particles are transported in a non-microtubule-dependent manner. In addition, 200 nm particles but not 500 nm particles accumulate in late endosomal or lysosomal compartments (27). Therefore, nanoparticles with a size close to 200 nm would be ideal for immunomodulatory properties, as cargo movement through the late endosomal compartment via intracellular endosomal receptors is thought to be a crucial step for engaging both adaptive and innate immune processes (28).

In summary, these findings suggest that size can not only determine how the nanoparticles distribute at an organ level, but also how they are internalized by cells and transported within cells. It is worth keeping in mind that the “ideal” size would likely vary for any given application in order to maximize circulation while achieving the desired biodistribution. For instance, in a meta-analysis that examined nanoparticle delivery efficacy to tumors covering over 100 studies from 2005 to 2015, it was found that smaller nanoparticles with hydrodynamic diameters below 100 nm have improved delivery efficacy (i.e., increased tumor accumulation and penetration) in comparison to particles >100 nm (29). Conceivably, the enhanced efficacy of tumor delivery is due to the ability of smaller nanoparticles to reach smaller blood and lymph capillaries and to extravasate at sites of endothelial compromise, such as the inflamed endothelium of cancers. While this analysis focused on nanoparticle delivery to cancer cells, the cancer milieu shares many cellular, and molecular hallmarks that are common to chronic inflammatory disease states, such as those in transplantation (30, 31). Overall, available literature suggests that particles larger than 1,000 nm exhibit toxicity secondary to risks of capillary obstruction, whereas particles smaller than 10 nm exhibit toxicity secondary to inefficient cellular clearance and prolonged cellular accumulation. It is important to recognize that while many *in vitro* and *in vivo* studies have determined an efficient size range for their specific study purpose, the heterogeneity in experimental parameters prohibits adequate comparisons between disparate nanoparticles. The field should therefore improve to consistently analyze the toxicity of new materials in parallel to investigating their new applications.

## Nanoparticle Composition for Immune Modulation

The next variable in determining how a nanoparticle might affect host inflammation is the nanoparticle composition.

Polymeric nanoparticles include those made from synthetic polymers such as PLGA [ploy(lactic-co-glycolic acid)], PLA (polylactic acid), chitosan, gelatin, polycaprolactone, and poly-alkyl-cyanoacrylates. The advantage of these types of polymeric materials for nanoparticles lies in their long shelf life and their enhanced ability for drug delivery: the process of their synthesis readily permits encapsulation of a wide range of hydrophobic and hydrophilic compounds as well as proteins (32, 33). In addition, by manipulating the proportion of their individual components, these synthetic polymers can be further tuned to have exquisite control over release of their encapsulated payload for the desired treatment duration. However, synthetic polymers have an intrinsic tendency to induce inflammation within the immediate microenvironment, due either to their lack of degradation or to their degradation products. For example, PLGA degrades to its based monomers, lactic acid and glycolic acid, leading to a decrease in the ambient pH which in turn promotes inflammation. The decrease in pH may also limit its capacity for loading certain drugs or bioactive molecules (34), although addition of other compounds such as alginate, chitosan, pectin during the encapsulation process may mitigate this problem. Despite this, concerns for toxicity of PLGA are overall low given that these two degradation products are naturally found in the body, albeit in small amounts, and are in general well-tolerated. This notion is supported by studies demonstrating that significant inflammation by PLGA particles is not observed even in exquisitely sensitive glial cells *in vitro* (35) or in the bronchial lavage from mice *in vivo* (36). Chitosan, another synthetic polymer, is less frequently utilized as a drug delivery carrier, due in part to its slow degradation that could lead to its accumulation upon repeated administration (37). However, chitosan is a cationic biopolymer and has the ability to form pores on the membrane of bacteria and fungi, conferring it with antibacterial and antifungal properties. The cationic charge also induces a strong mucoadhesive effect that confers it with permeability through tight junctions, allowing delivery of payload across mucosal membranes (38). Furthermore, under slightly acidic conditions, this material can be protonated to allow it to escape the endosomal space, increasing the cytosolic availability of its payloads (39, 40). While this ability by itself has not been shown to directly increase or reduce inflammation, through further encapsulation of anti-inflammatory payloads this platform can be used to increase the efficacy of these compounds due to endosomal escape (41–43). As such, chitosan-based particles are especially useful for cancer drug delivery (44), as the acidic tumor microenvironment is ideal for endosomal escape of chitosan nanoparticles. Another synthetic compound gelatin has also gained traction for use in nanoparticle technologies due to its biocompatibility, although its capability as a drug delivery vehicle is only beginning to be explored. In contrast, PLA, a plastic that has been explored as a possible nanoparticle material, shows significant toxicity from its lack of degradation (45, 46). The same is true with propylactone (47). Overall, optimization of polymer synthesis, functionalization and safety profiles would significantly broaden their use in the future due to their abundance and the ease of control of their fabrications (48).

Alternative to polymer-based nanoparticles is lipid-based nanoparticles. Lipid-based nanoparticles carry distinct advantages over polymeric or synthetic nanoparticles, partly because lipids are a natural component of cell membranes and therefore are less likely to induce inflammatory reactions. Liposomes have been successfully employed as drug delivery vehicles due to their high degree of biocompatibility (49) which lowers the barrier of entry in comparison to synthetic polymeric particles. This claim is supported by a long list of liposome-based platforms currently used in clinical practice (50). Lipid-based formulations began to show promise in 1995 with the development of the PEGylated liposomal formulation Doxil® for cancer therapies. The chemical characteristics of lipids, such as fatty acid chain length and saturation, can determine both the rigidity of the lipid bilayer as well as its intrinsic signaling properties. This can impact the particles' ability to diffuse through the extracellular matrix (51). Moreover, lipid composition has a marked impact on the protein corona of these particles through the charge of phospholipid heads at physiological pH. Specifically, cationic lipids attract negatively charged proteins, whereas anionic lipids attract positively charged proteins (52). Furthermore, cationic lipids are regularly used as toll-like receptor agonists or as adjuvants for vaccines (53). Consequently, reports have emerged demonstrating immune activation, especially by PEG-coated nanoparticles (54), via activation of pro-inflammatory pathways such as TLR4/MD2 and pro-apoptotic pathways such as CD14 (55). The opposite effect can also be achieved with anti-inflammatory lipids such as omega-3 polyunsaturated fatty acid (PUFA), lipoxins, aspirin-triggered lipoxins, resolvins, and protectins. These lipids have shown efficacy in reducing inflammation (56). Omega-3-PUFA reduces neutrophil infiltration and reactive oxygen species, accompanying an overall reduction in the magnitude of inflammatory response (57, 58). The immunomodulatory effects of omega-3-PUFA have been further demonstrated in animal models of stroke (59) and Alzheimer's disease (60). While the beneficial anti-inflammatory effects of PUFAs are well-documented, only limited studies have attempted to incorporate these lipids into the lipid bilayer of liposomes or lipid-based nanoparticles. Additional anti-inflammatory or immunomodulatory lipid-based signals have also not been explored to enhance the immune tolerogeneicity of nanoparticles. Future studies altering lipid compositions for signaling via specific molecular pathways to further reduce inflammation will have significant implications for the desired immune engineering. Furthermore, novel approaches for their surface modifications with engineered peptides (61, 62) and more recently with whole membrane proteins (31, 63, 64) will provide additional avenues for this platform to delivery specific antigens for the desired antigen-specific immune tolerance.

## Nanoparticle Surface Charge for Immune Modulation

The most important determinant of particle-host interaction at the cellular level is the surface chemistry of nanoparticles. In this context, an important parameter is the overall particle charge. From a formulation standpoint, it is simpler to fabricate nanoparticles that are either highly electronegative or electropositive, as this will ensure a monodisperse solution of particles and prevent particle aggregation. However, for considerations of safety and biocompatibility, nanoparticles must be close to neutral surface charge. Small deviations toward either a slight positive or a negative surface charge have a significant effect on their interaction with host cells, including their affinity for internalization and route of intracellular transport. Cationic particles are most avidly engulfed by macrophages, followed by anionic particles, while neutral particles have the least affinity for phagocytic macrophages (65). It is also important to keep in mind that the rate of internalization of a given particle does not necessarily correlate with its ultimate efficacy. For example, studies of intestinal epithelial cells have found that cationic nanoparticles are rapidly internalized, yet their transplant across the epithelial monolayer is slow. In contrast, anionic nanoparticles possess the opposite characteristics and are more efficient in their transport across the epithelial layer (66). Therefore, surface charge may not only be manipulated to guide the kinetics of nanoparticle internalization, but also to guide the kinetics of their transport across a cellular layer, and to increase their contact with immune cells. Overall, affinity of nanoparticle internalization, based on surface charge, is a part of the biologic signature that is specific to each nanoparticle and is further linked to surface opsonization of proteins (4). It highlights the potential advantages of using biomimetic approaches to functionalize whole cell membrane lipids and proteins to replicate the more complex interactions at the nanoparticle-cell interphase. In this context, it has been suggested that lipoprotein nanoparticles have superior bio-compatibility and biodegradability, in addition to their greater potential for further surface modifications (5).

One such strategy for further nanoparticle surface modification is the so called “bottom-up” approach. This approach employs well-defined chemical modifications of synthetic platforms to incorporate individual molecule/signal to the surface of lipid-based nanoparticles. The advantage of this approach is the total control over synthesis, but the disadvantage is the limited number of molecular signals that can be incorporated at one time. A common modification using this approach is polyethylene glycol (PEG) functionalization, which improves the biodistribution of nanoparticles by limiting their macrophage uptake. For example, a direct correlation has been established between PEG surface density and macrophage uptake (67). In this case, the efficacy of PEG correlated to its ability to limit contact between macrophages and the modified nanoparticles, highlighting the importance of contact-dependent internalization of nanoparticles by phagocytes.

## Nanoparticle Shape and Rigidity for Immune Modulation

Injectable nanoparticles are manufactured in a wide variety of shapes (e.g., spherical, cubic, rod, needle, plate-like shape, etc.). This can affect nanoparticle circulation time, biodistribution, and immune targeting. The most common nanoparticle shape is spherical which requires the lowest free energy of assembly, particularly when synthesis methodology employs a self-assembling process (68). Spherical nanoparticles may persist longer in the circulation due to their tendency to localize to the site of the fastest flow. This positions spheroids toward the center of the blood vessel, permitting minimum nanoparticle interaction with the vessel wall (15). Therefore, the tendency of spherical nanoparticles to marginate in the bloodstream is low. In terms of biodistribution, spherical particles exhibit homogeneous distributions in most organs, although with some preferential accumulation in the lungs, liver, and spleen (68). Non-spherical particles with higher aspect ratios (cylinders, rods) tend to exhibit a higher rate of cellular internalization (68). This is correlated with higher surface areas that trigger phagocytic activation of the interacting cells and can result in altered accumulation in organs enriched in phagocytes (3, 15). In this context, nanoparticle morphology may be further exploited for enhanced targeting of innate immune cells such as macrophages. For example, micelles and filomicelles have been shown to preferentially associate with liver macrophages and circulating phagocytes. In contrast, non-micelle vesicle morphologies have been shown to be more efficient at targeting splenic dendritic cells (2).

Nanoparticle toxicity is also significantly influenced by its shape and consequently its rigidity. For instance, needle-like particles are rigid and have been shown to directly cause damage to cell membranes upon contact (69) and may further cause activation of the inflammasome in immune cells. Other more exotic shapes such as prisms, nanorods, and nanoflowers made from gold have shown cellular toxicity *in vitro* (19). Nanoparticles engineered from gold, porous silicon, or hydroxyapatite tend to be most rigid, and are more likely to induce membrane damage during internalization. Rigidity also impacts biodistribution, as rigid particles tend to be subjected to faster first-pass clearance upon intravenous injection (70). On the contrary, “soft” nanoparticles, such as liposomes or other polymer-based nanoparticles, are not as prone to inducing cell membrane damage, and may be engineered to form less toxic non-spherical structures such as disc, cylindrical, and elongated rod-like micelles (71).

## Nanoparticle Cargo Encapsulation for Immune Modulation

There are a number of advantages of delivering payloads by their encapsulation within nanoparticles. This process limits susceptibility of cargo to biodegradation by host proteases or nucleases and increases their circulating half-life, thereby protecting the cargo from the extracellular milieu prior to reaching the intended target tissue. Encapsulation may also serve to protect off-target tissues from toxicity. A notable example of this latter principle is the encapsulation of anti-cancer compound doxorubicin within liposomes to reduce cardiac toxicity (72). This is because liposome formulations often exit where capillaries are disrupted by inflammation such as in tumor tissues, thereby preferentially targeting these sites (73). Because of this affinity for sites of inflammation, encapsulated cargo has significant potential to target immune cells for immunomodulation. Under non-inflammatory conditions, the route of administration may be leveraged to target the immune system. For example, subcutaneous injection permits transport to draining lymph nodes for delivery to resident immune cells. Encapsulation approaches are also compatible with multiple therapeutic cargos. For example, co-encapsulation of pro-apoptotic ABT-737 and camptothecin in PLGA-PEG nanoparticles was shown to exert a synergistic anti-tumor in experimental animals, while reducing toxic side effects of both compounds (74). A similar protective synergism has been reported in co-encapsulation of 6-mercaptopurine and daunorubicin for treating lymphoma (75). Encapsulation of therapeutic molecules can also improve drug bioavailability. For instance, curcumin and resveratrol are compounds shown to have anti-cell proliferative potential but have limited clinical efficacy due to their poor bioavailability (76). However, liposome formulations of these compounds aid in their improved pharmacokinetics and pharmacological efficacy (77). In the case of immune tolerance, nanoparticle-encapsulated rapamycin but not free rapamycin has been shown to induce immune tolerance when co-administered with antigen (78). The potential of nanoparticles for the goal of immune tolerance is further discussed below in the section titled “Biomimetic nanoparticles for immune tolerance.”

## Section 2. Biomimetic Nanoparticles for Immune Tolerance

The cutting edge of nanomedicine has turned its focus in recent years toward the design of “biomimetic” nanoparticles. “Biomimetic” nanoparticles, as the term implies, are nanoparticles designed based on biological systems and processes. Specifically here, we use this term to refer to nanoparticles that incorporate on their surface whole cell membranes or membranous proteins, which are derived from harvested cells, tissues, blood, or other biological sources. The application of this term may be further extended to nanoparticles that are functionalized with recombinant proteins (79, 80), or extracted cell membrane proteins (63, 81). Such surface modifications endow the nanoparticles with sophisticated cellular functions, such as adhesion (29, 82, 83), signaling (31), and transmigration. In addition, they have been exploited to target the coated nanoparticles to antigen-specific immune cell subsets (84). This effectively mimics the function of the cells from which the membrane proteins are derived, all the while retaining drug-loading capacities. The ability of biomimetic nanoparticles to activate the immune system has been extensively exploited in the field of vaccine design with the goal to increase vaccine-specific immune responses (79, 80, 85, 86). Many of the same principles employed in vaccine designs, such as specific tissue targeting and antigen presentation, are similarly applicable to strategies of immune tolerance.

## Two Approaches for Assembling Biomimetic Nanoparticles

Currently, there exist two validated approaches for assembling biomimetic nanoparticles. The first approach is coating a polymer core with whole membrane proteins derived from cells or cell ghosts (87). This strategy has the benefit of preserving the chemical properties of the core polymer (e.g., drug loading, drug release rate, etc.) while acquiring all surface proteins that are required for achieving specific biological purposes (e.g., prolonged circulation time and increased biocompatibility, etc.) (88). The second approach is functionalizing the lipid bilayer of liposomes with extracted whole membrane proteins derived from cells or cell ghosts. This approach has been proven effective in simultaneously incorporating a large number (over 300) of membrane proteins extracted from immune cells such as macrophages (63, 81). Both approaches of biomimetic design represent a significant increase in the capabilities of protein functionalization. Prior to this, nanoparticles, whether made from lipids or polymer materials, were only functionalized through a handful of proteins or peptides. As such, this type of functionalization has been termed as a “top down” approach.

Biomimetic approaches are effective for sophisticated tissue-specific targeting that closely mimics biological processes, particularly when considering the pathophysiology of disease states. One such example is to functionalize liposomes with membrane proteins from leukocytes to specifically target to activated endothelium (63, 64, 89). These particles, called “leukosomes,” incorporate the constellation of proteins that are naturally present on leukocytes. This includes leukocyte ligands such as LFA-1, CD45, CD47, PSLG-1, and Mac-1, which can confer a capacity to target specific cell types. In a state of localized or systemic inflammation (such as seen in transplantation), the endothelium is often activated, which leads to a compromise in its integrity and subsequently an increase in inflammatory cell adherence and permeability (30). Leukosomes functionalized with membrane proteins have shown a 4-fold increase in efficacy in targeting inflamed endothelium (31, 63) in comparison to bare liposomes (64). This platform has been shown to have intrinsic anti-inflammatory or immunomodulatory properties manifested in their ability to mitigate inflammatory bowel disease (90) as well as sepsis (31). For instance, study in a sepsis model has shown that a reduction of inflammation by leukosomes is mediated through their interaction with macrophages, which induces a decrease of expression of pro-inflammatory genes (IL-6, IL-1beta, and TNF-α) and an increase of expression of anti-inflammatory genes (IL-10 and TGF-β).

Generally, these particles also retain their drug loading capacities which are useful for targeted drug delivery. For example, liposomes assembled with membrane proteins from mesenchymal cells to create “nanoghosts” have been used for encapsulation of soluble TNF-related apoptosis-inducing ligand (sTRAIL), a molecule known for its short circulation time and hepatotoxicity. Such sTRAIL encapsulating nanoghosts exhibit increased circulation time and reduced hepatotoxicity, and selectivity target certain cells with cytosolic and nuclear accumulation (90). These leukosomes have also been successfully employed to target activated endothelium in cancers and atherosclerosis (30). For instance, rapamycin encapsulating leukosomes have been shown to reduce inflammation in mouse models of atherosclerosis (89).

Another example of the biomimetic approach is the functionalization of a polymer core with cell membrane proteins to enhance nanoparticle targeting capabilities (87). This strategy has been successfully employed to create nanoparticles composed of a polymer core and a platelet membrane envelop. As such, these nanoparticles effectively mimic the function of platelets, with selective adhesion to damaged vasculatures as well as enhanced binding to platelet-adhering pathogens. This approach has been successfully employed as a treatment for microbial infections as “nanosponges” that bind to infectious agents and absorb pathogen secreted toxins (6). Even the most straightforward use of these particles as a drug delivery agent has been shown to increase the efficacy of antibiotics (91). Despite surface functionalization of particles with cell membrane proteins, such biomimetic platforms usually allow retention of physiochemical properties of the core polymer, such as prolonged release kinetics and/or pH-sensitive release of cargos. Additional advantages of these platforms include the ability to incorporate self-recognition markers (90, 92).

While the advantages are clear, the feasibility of biomimetic approaches is somewhat limited by the membrane functionalization step, as whole cell membranes are not simple to obtain or manipulate, and are generally unstable. Regardless, pioneering studies in small animal disease models have demonstrated successful translation of these platforms to therapies for cancers (93) and infectious diseases (91, 94). In theory, the biomimetic design would allow functionalization of nanoparticles with membranes or membrane proteins from any cell in the body. Such versatility would also confer these nanoparticles with the ability to replicate the function of any cell in the body. Testing the full potential of this platform is an exciting area of new research and will undoubtedly lead to novel therapies to be formulated and manufactured, including for antigen presentation and for immune tolerance.

## Biomimetic Nanoparticles for Antigen Presentation

A unique property of biomimetic nanoparticles that has been captured for modulating immunological responses is that functionalization of proteins on nanoparticle surfaces in general increases the stability of proteins in comparison to that of soluble proteins or cell lysates. This advantage has been employed for vaccine development for sustained antigen delivery. In a study using a high density lipoprotein (HDL) mimetic “nanodisc” coupled with antigenic peptides, improved antigen delivery to lymphoid organs and sustained antigen presentation by antigen presenting cells (APCs) are observed. Consequently, this platform elicits up to a 47-fold greater frequency of antigen-specific cytotoxic T cells (CTL) than by similar peptides (79). In addition, HDL-based nanodiscs are comparatively easy to synthesize, as various lipid-based antigens can be readily incorporated onto the surface through simple mixing. This advantage has allowed inclusion of both major histocompatibility complexes (MHCs) and tumor antigens to the nanoparticles, and together with immune-blockade treatment, has led to 90% of mice free of tumors in one study (79). This advantage will significantly increase the value for the clinical application of this platform.

The ease of synthesis and functionalization of various antigens to the surface is not exclusive to the nanodisc platform, and has also been experimented for other liposomal particles. Since these particles contain a lipid bilayer that is similar to that of cells, it is possible to insert membrane and transmembrane proteins into their lipid bilayer. While it is possible to synthesize biomimetic liposomal particles using the well-stablished thin layer evaporation (TLE) and extrusion approach, the introduction of microfluidic synthesis procedures has greatly facilitated introduction of a wider range of proteins and protein sources to the liposomal particle manufacturing process. In contrast to the physical extrusion needed to create homogeneous liposomes in the common TLE approaches, microfluidic synthesis relies on micromixing that preserves the structure of complex proteins by subjecting source proteins to only low shear, low heat, and low pressure (81). This synthesis procedure has allowed successful functionalization of whole membrane proteins from red blood cells, leukocytes, platelets, cancer cells, among others. In a study using biomimetic lipid nanoparticles functionalized with proteins derived from activated T cells to target inflamed bowels to treat inflammatory bowel disease, a significant reduction in key inflammatory markers (IL-6 and TNFα) and restoration of colon tissue architecture has been observed (95). The “specialized leukosomes” (SLK) used in this study did not contain a pharmacological payload, suggesting that the mechanism of action of SLK is simply due to its surface functionalization with T cell membrane proteins.

Polymer-based nanoparticles have also been exploited for antigen presentation. There, antigens are usually delivered as surface-linked peptides. These nanoparticles are then internalized by dendritic cells which in turn cross-present the delivered peptide antigens via their MHC molecules to elicit host CD8^+^ and CD4^+^ T cell responses. Many studies of using this type of nanoparticles for antigen presentation have focused on targeting lymph nodes, based on the rationale that a high density of antigen present cells is present at this location. Smaller nanoparticles (<30 nm) are capable of draining in lymphatic capillaries in a size-dependent fashion (96). In one study, 25 and 100 nm nanoparticles were tested, with a much higher accumulation in dendritic cells seen with the smaller 25 nm particles (97). Based on these results, small nanoparticles can be employed to specifically target lymph nodes for antigen presentation.

While most of the approaches available in the literature have focused on using polymeric particles to induce an adjuvant-like response, these types of particles coupled with surface membrane protein functionalization could potentially be used to induce tolerance in an antigen-dependent manner in both transplant and autoimmune diseases. One advantage of employing nanotechnology for antigen presentation is the possibility of simultaneous functionalization of a variety of antigens or antibodies on the surface of nanoparticles. This advantage is further enhanced by recent advancements in synthesis procedures that allow incorporation of the complete set of cell membrane proteins to the surface to create true biomimetic nanoparticles. While a great deal has been learned using nanotechnology for vaccine development, knowledge in incorporating specific molecules to target APCs and to induce inflammatory responses could very well be adapted to reduce or control them for application toward transplantation and autoimmune diseases.

## Biomimetic Nanoparticles for Immune Tolerance

To date, the goal of complete immune tolerance to organ and stem cell allografts, remains elusive. For this to be achieved, tolerance to alloantigens by nanoparticle strategies must be antigen-specific and sustainable. This is in contrast to current standards of care that often rely on chronic and generalized immunosuppression, the unintended consequences of which include increased susceptibility to infections and malignancies. In the setting of allograft transplantation, nanoparticle approaches for achieving operational tolerance must target the cross-talk between innate and adaptive arms of immunity. For example, direct presentation of donor MHC may trigger allorecognition and activation of host cytotoxic T cells. In addition, host effector T cells may also be activated through interactions with host APCs, particularly dendritic cells that cross-present donor antigens within recipient MHCs. This leads to the recruitment of donor-reactive CD4^+^ and CD8^+^ T cells to allografts and promotes graft rejection (98, 99) or chronic allograft vasculopathy (7). In addition to MHCs, donor ABO blood-group and other minor alloantigens may also serve to activate the adaptive immune response. These aforementioned molecules represent key alloantigen targets for strategies of nanoparticle-mediated antigen-specific tolerance.

In this setting, professional APCs such as dendritic cells regulate immune activity and program the fate of the interacting T cells toward activation, anergy, or deletion. APCs can promote reactivity to alloantigen in the setting of ligands that activate APC pattern recognition receptors (PRR) (100, 101), nucleotide-binding oligomerization domain-like receptor (NLRs), or C-type lectin receptors (102, 103). Thus, inhibition of these APC receptors is a logical target of nano-platforms for immune tolerance. Another approach is prophylactic exposure to alloantigen in a non-inflammatory milieu. For example, nanoparticle-mediated administration of allo-antigens in combination with anti-inflammatory compounds may be an effective therapeutic strategy. Candidate anti-inflammatory nano-platforms include citrate-coated nanoparticles with anti-oxidative and anti-inflammatory properties (13), carbon nanotubes which have been shown to inhibit the activation of lung-resident APCs (104), nanoparticles co-delivering an antigen and a tolerogenic molecule aryl hydrocarbon receptor (AhR) ligand to induce tolerogenic DCs (105), and nanoparticles coated with antigenic peptides bound to MHC II molecules to generate and expand antigen-specific regulatory T cells (106). An additional interesting approach here is to take advantage of the role of chromatin in the maintenance of natural immune tolerance and create DNA-protein nanocomplexes that can effectively induce immune tolerance to the complexed protein antigens (107). However, while successful in several immune disease models of model antigens, this approach has yet to be tested for transplantation tolerance. An alternative approach is to sequester inflammatory signals through surface ligands that act as a molecular “sponge.” Lastly, inhibitory decoy targeting of costimulatory signals on the surface of T cells also has the potential to impair T cell activation. A summary of recent notable experimental immunoregulatory nanoparticles for transplantation tolerance is presented in Table 1.

**Table 1:** Recent notable experimental immunoregulatory nanoparticles for transplantation tolerance.

**Nano platform**	**Graft or target**	**Immune modulation and outcome**	**References**
Biodegradable poly(lactide-co-glycolide) (PLG) particle with donor peptides	Skin graft mouse model	Expansion of graft-infiltrating T-regulatory cells and graft prolongation	(108)
Mycophenolate Mofetil-loaded Copolymer PEG-PLGA nanoparticles	Donor organ prior to transplant	Suppressed intragraft pro-inflammatory cytokines and chemokines and reduced cardiac transplant vasculopathy in mouse model	(109)
High-density lipoprotein nano-platform encapsulated with mTOR or TRAF6 inhibitors	Vascularized organ in mouse model	Regulatory macrophage phenotype and regulatory T cell expansion leading to indefinite allograft survival	(110)
Monoclonal antibody-coated microparticle carrying tacrolimus and anti-CD3	Lymph nodes	Elevation of intragraft Tregulatory cells and prolonged heart allograft in mouse model	(111)
Positively charged polymer to interacts with negatively charged siRNA cargo	MHCII reduction of graft arterial endothelial cells	Reduction of allogenic T-cell response to human graft in moues model	(112)
Biomimetic PLGA coated with donor cell lysates	Allogeneic islet cell transplant	Graft prolongation in mice	(113)

Additional strategies for reprograming antigen-exposed dendritic cells for immune tolerance is the co-administration of Vitamin D, dexamethasone, or anti-inflammatory cytokines such as IL-10 (114). The administration of Vitamin D in the form of 1,25(OH)2D3 to monocyte-derived dendritic cells (D3-DCs) reduces MHC class II and co-stimulatory molecule expression, while increasing IL-10 expression. These D3-DCs prevent priming of naïve CD4^+^ or CD8^+^ T cells, instead induce apoptosis of effector T cells and promote differentiation of antigen-specific regulatory T cells (Tregs) from naïve CD4 T cells. Similarly, dexamethasone is also capable of inducing a tolerogenic phenotype in dendritic cells, characterized by a sustained high level of IL-10 production but suppressed pro-inflammatory IL-12 and IFN-γ production. Another well-established function of dendritic cells is their role in the differentiation of CD4^+^ T cells into Tregs (115, 116). For example, rapamycin-exposed DCs induce expansion of CD4^+^CD25^bright^ Tregs that are capable of suppressing antigen-stimulated proliferation of naive CD4^+^ T cells. Thus, incorporation of these immunoregulatory compounds into nanoparticle platforms that target dendritic cells, has the potential to tailor desirable T cell responses.

In the case of transplantation tolerance, the spectrum of alloantigens delivered must be sufficiently inclusive to modulate subsequent immune responses from a solid organ or stem cell transplant. In this setting, it is unlikely that an individual or a single class of MHCs will confer complete tolerance (117). This is not a new concept, as the development of vaccines is also reliant on broad antigen presentation to mount a complete protective response. In contrast to vaccines, fewer studies have been conducted on antigen-specific modulation of dendritic cells for transplant tolerance. Current approaches in pre-clinical models use cell lysates or apoptotic cells of donor origin to incorporate the full spectrum of potential donor alloantigens (118, 119). However, such cellular approaches are often hindered by expensive and time-consuming scale-up operations that render their clinical applications less practical. In this respect, nanotechnology is uniquely positioned to address this limitation by engineering approaches that will: (1) incorporate a complete spectrum of donor antigens on the delivery platform and (2) incorporate a wide variety of surface proteins that will ensure their delivery to the intended anatomic or cellular location, and elicitation of the desired immune tolerance response.

## Concluding Remarks

Taken together, we have reviewed and postulated a series of design principles that are options for flexible nanoparticle bioengineering. With this wide variety of nanoparticle platforms, the future is bright for new research and applications for the purpose of targeting the immune system for immune tolerance.

## Author Contributions

ET, CB, CJ, and XL wrote the manuscript. XL and ET edited and finalized the manuscript.

## Conflict of Interest

The authors declare that the research was conducted in the absence of any commercial or financial relationships that could be construed as a potential conflict of interest.
